# Regulated reconstitution of spindle checkpoint arrest and silencing through chemically induced dimerisation *in vivo*

**DOI:** 10.1242/jcs.219766

**Published:** 2018-10-04

**Authors:** Priya Amin, Sadhbh Soper Ní Chafraidh, Ioanna Leontiou, Kevin G. Hardwick

**Affiliations:** Institute of Cell Biology, School of Biological Sciences, University of Edinburgh, King's Buildings, Max Born Crescent, Edinburgh, EH9 3BF, UK

**Keywords:** Mps1, Checkpoint, Dimerisation, Mitosis, Reconstitution, Spindle

## Abstract

Chemically induced dimerisation (CID) uses small molecules to control specific protein–protein interactions. We employed CID dependent on the plant hormone abscisic acid (ABA) to reconstitute spindle checkpoint signalling in fission yeast. The spindle checkpoint signal usually originates at unattached or inappropriately attached kinetochores. These are complex, multiprotein structures with several important functions. To bypass kinetochore complexity, we took a reductionist approach to studying checkpoint signalling. We generated a synthetic checkpoint arrest ectopically by inducing heterodimerisation of the checkpoint proteins Mph1 (the fission yeast homologue of Mps1) and Spc7 (the fission yeast homologue of KNL1). These proteins were engineered such that they cannot localise to kinetochores, and only form a complex in the presence of ABA. Using this novel assay we were able to checkpoint arrest a synchronous population of cells within 30 min of ABA addition. This assay allows detailed genetic dissection of checkpoint activation and, importantly, also provides a valuable tool for studying checkpoint silencing. To analyse silencing of the checkpoint and the ensuing mitotic exit, we simply washed out the ABA from arrested fission yeast cells. We show here that silencing is critically dependent on protein phosphatase 1 (PP1) recruitment to Mph1-Spc7 signalling platforms.

## Abstract (precis)

**Summary:** Abscisic acid is used as a chemically induced dimeriser (CID) to bring together Mph1 kinase and Spc7 and initiate spindle checkpoint signalling *in vivo*. Simply washing out the abscisic acid enables checkpoint silencing to be studied.

## INTRODUCTION

Spindle checkpoint signalling was initially reconstituted in *Xenopus* egg extracts (Kulukian et al., 2009; Minshull et al., 1994) and most recently using recombinant complexes of human checkpoint proteins (Faesen et al., 2017). Major advantages of such *in vitro* assays are that complex systems can be simplified through biochemical fractionation and manipulated through immunodepletion. They also enable the regulated addition of specific components, whereby the timing, concentration and activity of these can all be varied.

In parallel, yeast genetics has driven the identification of most of the molecular components of this pathway, such as the mitotic arrest deficient (Mad) and budding uninhibited by benzimidazoles (Bub) proteins (Hoyt et al., 1991; Li and Murray, 1991) and their Cdc20 effector (Hwang et al., 1998; Kim et al., 1998). This combination of yeast genetics and *in vitro* reconstitution has proven invaluable in dissecting the molecular mechanism of action of spindle checkpoint signals and inhibition of the downstream effector Cdc20-APC/C (London and Biggins, 2014; Musacchio, 2015).

Here, we have employed a hybrid approach, using yeast genetics and partial reconstitution of the pathway *in vivo*. We used synthetic biology to re-wire and simplify the upstream part of the checkpoint signalling pathway and chemically induced dimerisation (CID) to add an extra level of regulation that can be easily controlled experimentally in intact cells. Employing this strategy, we were able to achieve the following outcomes. (1) We simplified the system through regulated, ectopic activation of the spindle checkpoint, enabling kinetochore-independent studies. (2) We used yeast genetics to enable rapid iterative analyses. (3) We employed synthetic biology and CID to generate specific complexes in an experimentally controlled fashion. (4) We used abscisic acid (ABA) addition and wash-out to provide tight temporal control of the initiation and termination of checkpoint signalling.

More specifically, we generated a synthetic checkpoint arrest ectopically by inducing heterodimerisation of the checkpoint proteins Mph1 (the fission yeast homologue of Mps1) and Spc7 (the fission yeast homologue of KNL1) ^ ^in fission yeast. This led to checkpoint arrest in a synchronous population of cells within 30 min of addition of the plant phytohormone ABA. As expected, this checkpoint response required the downstream Mad and Bub factors. To analyse silencing of the checkpoint, we simply washed out the ABA from arrested cells and analysed mitotic exit. We found that the kinetics of release was critically dependent on recruitment of protein phosphatase 1 (PP1) to the Mph1-Spc7 signalling platform.

## RESULTS

We previously published a synthetic checkpoint arrest assay (SynCheck) in which we activated the spindle checkpoint in fission yeast using heterodimers of TetR-Spc7 and TetR-Mph1 kinase (Yuan et al., 2017). However, in those experiments, dimerisation was constitutive, being driven by formation of Tet repressor dimers (TetR). Thus, checkpoint signalling was challenging to regulate, both in terms of initiation and termination. We controlled checkpoint arrest at the transcriptional level using an *nmt* promoter to drive expression of the TetR-Mph1 fusion protein. Unfortunately, the fission yeast *nmt1* promoter requires induction in medium lacking thiamine for several hours. As a consequence, the peak of arrest was observed ∼14 h after induction and was not as synchronous as hoped. To improve both timing and control, we modified our approach by employing CID to give tight temporal control over the initiation and termination of checkpoint signalling.

### Generation of SynCheckABA

**Fig. 1.**
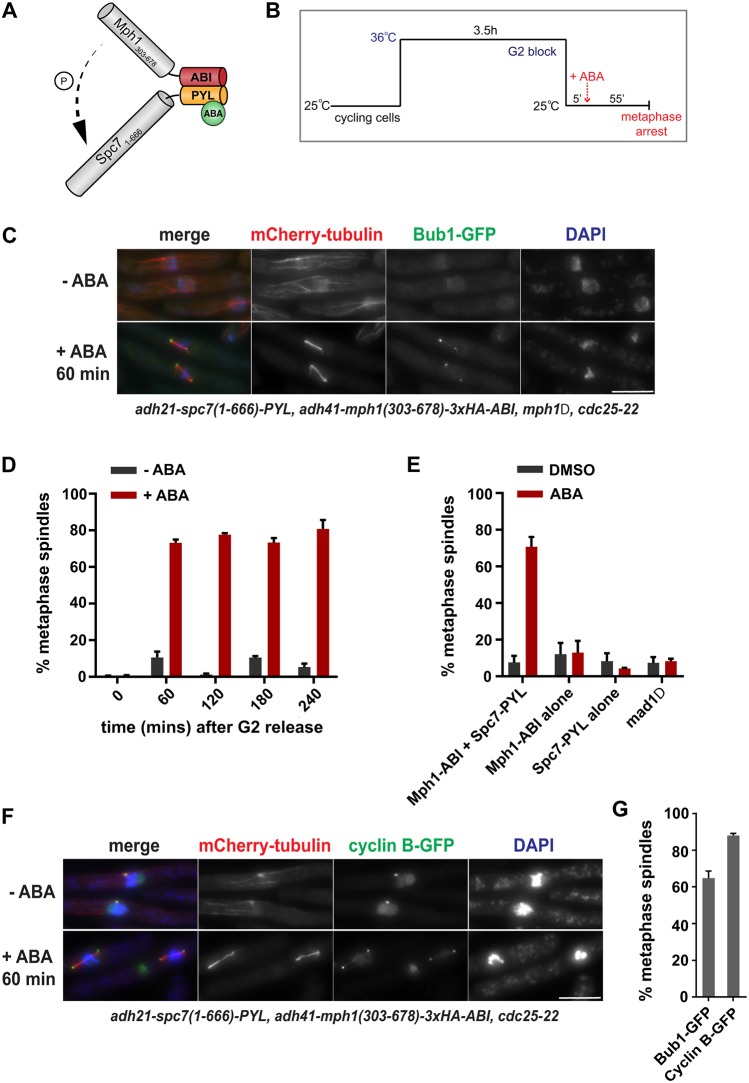
**Rapid induction of spindle checkpoint arrest using ABA for CID of Mph1-Spc7.** (A) Representation of the Mph1-Spc7 heterodimer induced by ABA addition. (B) Work flow of the pre-synchronisation in G2 (*cdc25-22*), followed by release into mitosis at 25°C and then induction of checkpoint arrest through the addition of ABA. (C) Fixed cell images taken of the arrested ABA-induced strain 60 min after ABA addition. Microtubules are seen in red (mCherry-Atb2), the checkpoint protein in green (Bub1-GFP) and chromatin in blue (DAPI). (D) Quantification of cultures (±ABA addition) through a 4 h time course after release from G2. Samples were fixed every 60 min and scored as metaphase arrested if they had short metaphase spindles and a single mass of condensed chromatin. More than 100 cells were analysed per strain at each time point. The experiment was repeated three times. (E) Quantification of the strains indicated at the 60 min time point after release from the G2 block (ABA added 5 min after release). *mad1Δ* is the Mph1-ABI Spc7-PYL strain with *mad1* deleted. Cells were scored as metaphase arrested as for D. At least 100 cells were analysed per strain at each time point. The experiment was repeated three times for each strain. (F) Fixed cell images taken of the SynCheckABA strain with Cdc13–GFP at spindle poles bodies 60 min after ABA addition. Microtubules are seen in red (mCherry-Atb2 is labelled fission yeast tubulin), cyclin B in green (Cdc13-GFP) and chromatin in blue (DAPI). (G) Comparison of ABA-induced metaphase arrest at 60 min for an Mph1-ABI Spc7-PYL strain containing Bub1-GFP or another Cdc13-GFP. This experiment was repeated twice. All data are plotted as mean±s.d. Scale bars: 10 μm

Following the strategy of Crabtree and colleagues (Liang et al., 2011), we fused the PYL domain (residues 33-209) of the ABA receptor after the N-terminal 666 amino acids of fission yeast Spc7. By deleting the C-terminal half of Spc7, this protein is unable to be targeted to kinetochores because it lacks the Mis12-interacting region (Petrovic et al., 2016; Petrovic et al., 2014). This fusion protein was expressed from the constitutive *adh21* promoter (Tanaka et al., 2009). The ABI domain (residues 126-423) of ABI1 was fused to the C-terminus of the Mph1 spindle checkpoint kinase. We also deleted the first 301 amino acids of Mph1 to prevent it going to kinetochores (Heinrich et al., 2012). This Mph1-ABI fusion protein was expressed from the adh41 promoter (Tanaka et al., 2009). In the presence of ABA, the PYL and ABI domains are sufficient to form a tight complex (Miyazono et al., 2009), thus forming complexes of Mph1-ABI and Spc7-PYL (Fig. 1A). We combined these constructs in a strain that also had the *cdc25-22* mutation, enabling synchronisation in G2, the Bub1 checkpoint protein tagged with GFP and microtubules labelled with mCherry-Atb2 (α-tubulin).

### Inducing Spc7-Mph1 heterodimers to trigger metaphase arrest

**Fig. 2.**
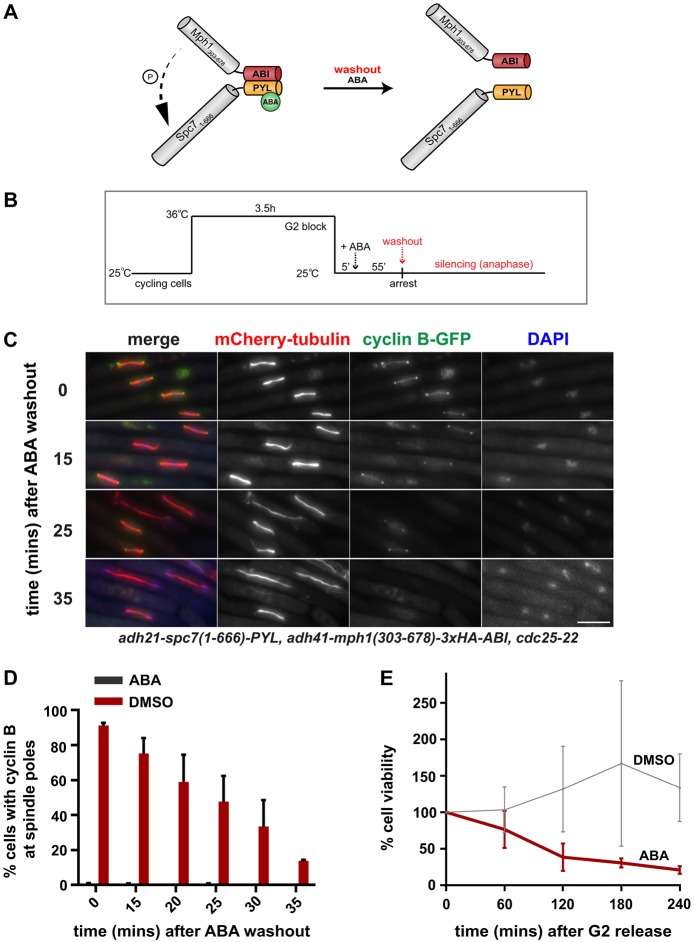
**Silencing of spindle checkpoint signalling after ABA wash-out.** (A) Representation of the dissociation of Mph1-Spc7 heterodimers after ABA wash-out. (B) Silencing work flow: pre-synchronisation in G2 (*cdc25-22*), induction of checkpoint arrest through the addition of ABA, subsequent wash-out of ABA 60 min later. (C) Fixed cell images taken of the arrested SynCheckABA strain at 0, 15, 25 and 35 min after ABA wash-out. Microtubules are seen in red (mCherry-Atb2), cyclin B in green (Cdc13-GFP) and chromatin in blue (DAPI). Scale bar: 10 μm. See Fig. S2B for an alternatively coloured version of similar images. (D) Quantification of Cdc13-GFP at spindle pole bodies in the SynCheckABA cultures (plus ABA or DMSO). Samples were fixed and scored for the presence of Cdc13 at spindle pole bodies. The +DMSO control did not arrest in metaphase. More than 150 cells were analysed per strain at each time point. This experiment was repeated three times. (E) The viability of SynCheckABA-arrested strains was determined by plating cells 0, 60, 120, 180 and 240 min after release from a G2 block, where DMSO or ABA was added 5 min after release from the G2 block. Cell viability over time was plotted as a percentage relative to that at time zero. Cells were plated in triplicate. The experiment was repeated three times. All data are plotted as mean±s.d.

Cells were synchronised in G2 using a temperature-sensitive *cdc25-22* mutant that blocks cells in G2 after 3.5 h at 36°C. When cells were shifted to 25°C, they were ‘released’ from the block, enabling progression through the cell cycle. After 5 min, ABA was added to activate the spindle checkpoint through the formation of Spc7-PYL and Mph1-ABI heterodimers (Fig. 1B). We observed that 60 min after ABA addition to the synchronous population of cells, over 70% of cells had short metaphase spindles (Fig. 1C,D). The metaphase arrest could be sustained for at least 4 h (Fig. 1D). We tested a range of ABA concentrations (0-500 µM) and found that 250 µM was optimal for reproducible, robust arrests (Fig. S1A). The ABA can be added later (e.g. 20 min after *cdc25* release) and cells arrest with similar efficiency to that observed after anti-microtubule drug treatment with carbendazim (see Fig. S1B). Without pre-synchronisation in G2, the mitotic index increases over time and reaches a peak 4 h after ABA addition (Fig. S1C). In our previous SynCheck studies, cells arrested for several hours but then died (Yuan et al., 2017). We wanted to determine whether the ABA arrest also had a significant effect on cell viability or whether our ability to release this arrest (through ABA wash-out) meant that viability was maintained. After ABA treatment, we found a gradual drop in cell viability (see Fig. 2E), which was similar to that observed upon anti-microtubule drug treatment (data not shown).

In the arrested cells, we observed Bub1 enrichment at the spindle poles (Fig. 1C). This is consistent with our previous SynCheck assay, where movement of all spindle checkpoint proteins to spindle poles was reported to be Mad1-Cut7 kinesin driven (Yuan et al., 2017). As expected, deleting the first N-terminal coiled coil (136 amino acids) of Mad1, required for its interaction with Cut7 (Akera et al., 2015), prevented Bub1 accumulation at spindle poles. This de-localisation of checkpoint proteins from spindle poles did not affect the efficiency of the arrest (Fig. S1D), as found in SynCheck (Yuan et al., 2017).

ABA-induced metaphase arrest is dependent on heterodimerisation of Spc7-PYL and Mph1-ABI. Strains lacking either the Mph1-ABI component or the Spc7-PYL component failed to arrest in the presence of ABA (Fig. 1E). Deleting the downstream checkpoint protein Mad1 abolished the arrest (Fig. 1E), showing that ABA-induced arrest is checkpoint dependent. In these constructs, Spc7 and Mph1 lack their kinetochore-binding domains, making initiation of this arrest ectopic and independent of the complexities of the kinetochore. The Mph1-ABI, Spc7-PYL strain used above lacks endogenous *mph1*, which prevents all Mad and Bub checkpoint proteins from targeting to kinetochores (Heinrich et al., 2012). As an additional measure, to confirm kinetochore independence, we employed a strain containing the *spc7-12A* MELT mutant allele (Mora-Santos et al., 2016; Yamagishi et al., 2012). This mutant Spc7 kinetochore component cannot be phosphorylated by Mph1, preventing recruitment of Bub3-Bub1, and thereby Mad1-Mad2 complexes, to kinetochores. The *spc7-12A* mutant arrested with very similar efficiency to *spc7+* cells under ABA control (Fig. S1F), indicating that the Spc7wt-PYL Mph1-ABI heterodimer does not need to be aided by endogenous kinetochore-based checkpoint signalling to generate a checkpoint arrest. Importantly, *spc7-12A-PYL* fusion protein was unable to generate an arrest in combination with Mph1-ABI, demonstrating that the ectopic signalling scaffold needs to be phosphorylated on conserved Spc7 MELT motifs to recruit Bub3-Bub1 complexes for active signalling (Fig. S1G).

Crucial consequences of checkpoint action are the stabilisation of cyclin B and securin. Using a modified strain, we analysed cyclin B (Cdc13) levels in the ABA-induced arrest. Fig. 1F shows that Cdc13-GFP accumulated on short metaphase spindles and was enriched at mitotic spindle poles, as expected. As a technical aside, we found that different tags can affect the efficiency of the ABA-induced arrest. For example, this Cdc13-GFP strain reproducibly arrests more efficiently than the strain containing Bub1-GFP (Fig. 1G). This is probably a result of a partial loss of function when C-terminally tagging the Bub1 checkpoint protein. The Cdc13-GFP strain also contains the endogenous wild-type *Mph1* gene, but we found that this did not significantly impact the efficiency of arrest (see Fig. S1E).

Thus, we have reconstituted a robust, kinetochore-independent checkpoint arrest that can be initiated very simply *in vivo* through ABA addition to culture media. This works efficiently in both minimal (PMG) and rich (YES) fission yeast growth media. Hereafter, we refer to this assay as SynCheckABA.

### A novel spindle checkpoint silencing assay

A significant advantage of SynCheckABA is the ability to reverse the effects of ABA by simply washing cells with fresh medium lacking ABA and thereby releasing them from metaphase arrest (Fig. 2A,B). We can use this to study spindle checkpoint silencing, which has proven to be technically challenging in the past. Fig. 2C,D demonstrates that washing out the ABA results in rapid cyclin degradation and spindle elongation (see also Fig. S2A).

### Regulation of spindle checkpoint silencing

**Fig. 3.**
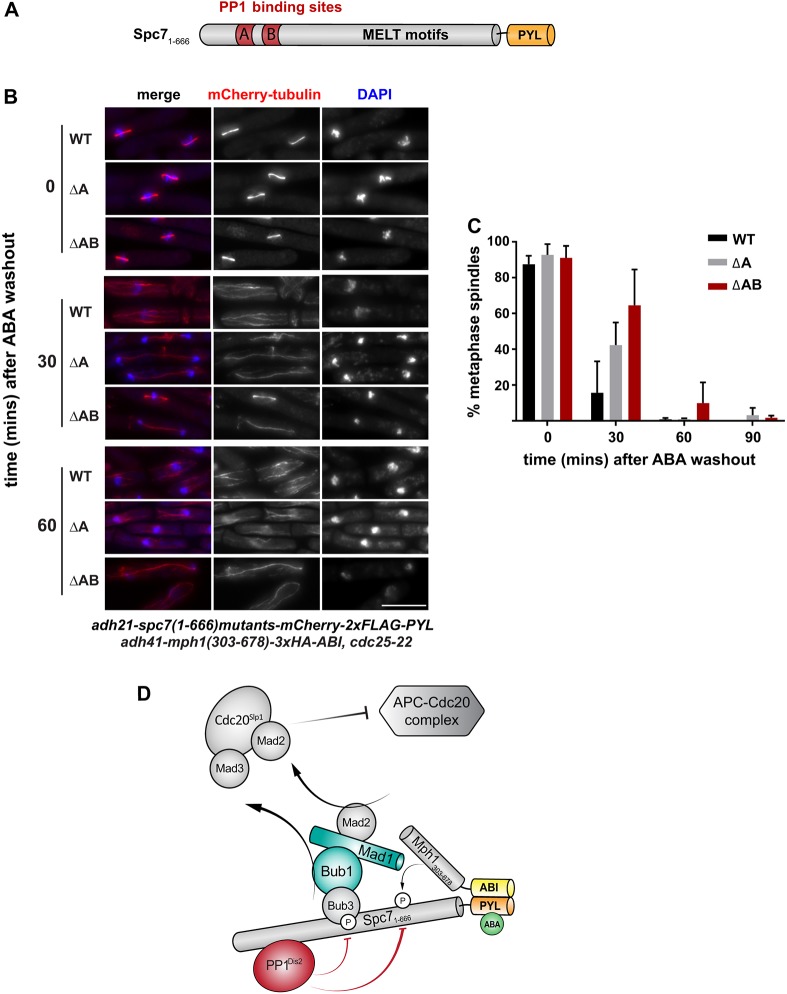
**Checkpoint silencing in SynCheckABA is dramatically slowed when the Spc7^KNL1^ binding sites for PP1^Dis2^ are deleted.** (A) Schematic of Spc7^KNL1^ indicating the N-terminal PP1-binding motifs (A motif, SILK; B motif, RRVSF). The MELT motifs form binding sites for Bub3-Bub1 complexes once they have been phosphorylated by Mph1 kinase. (B) Images of cells expressing wild-type Spc7_1-666_ (WT) or mutants with deletion of the A motif (ΔA) or both the A and B motifs (ΔAB). Time points were analysed at the time of ABA wash-out (time zero) and 30 and 60 min post-wash. Scale bar: 10 µm. See Fig. S3 for non-red-green colour scheme. (C) Quantification of release from checkpoint arrest in WT, ΔA or ΔAB strains. The experiment was repeated three times. More than 100 cells were analysed per strain at each time point. Data are plotted as mean±s.d. (D) Schematic of SynCheckABA: activating (Mph1) and silencing (PP1) factors bind nearby on the Spc7 scaffold. The balance of their activities determines how much MCC is generated and thus whether anaphase onset is inhibited.

Previous work has shown that PP1 (Dis2) is a key spindle checkpoint silencing factor in yeasts (Meadows et al., 2011; Pinsky et al., 2009; Vanoosthuyse and Hardwick, 2009). The N-terminus of Spc7 has two conserved motifs (SILK and RRVSF, also referred to as the A and B motifs) that mediate stable PP1 association (Fig. 3A). Mutation of both binding sites leads to a lethal metaphase block in *Saccharomyces* *cerevisiae* and *Schizosaccharomyces* *pombe* (Meadows et al., 2011; Rosenberg et al., 2011). There are additional kinetochore-binding sites for PP1 such as Klp5 and Klp6 (Meadows et al., 2011) and these are relevant to checkpoint silencing, although binding to Spc7 appears to be the major player. In human cells, similar motifs are found at the N-terminus of KNL1; PP1 binding is regulated by Aurora B activity as this kinase can directly phosphorylate the B motif, disrupting PP1 association (Liu et al., 2010).

**Fig. 4.**
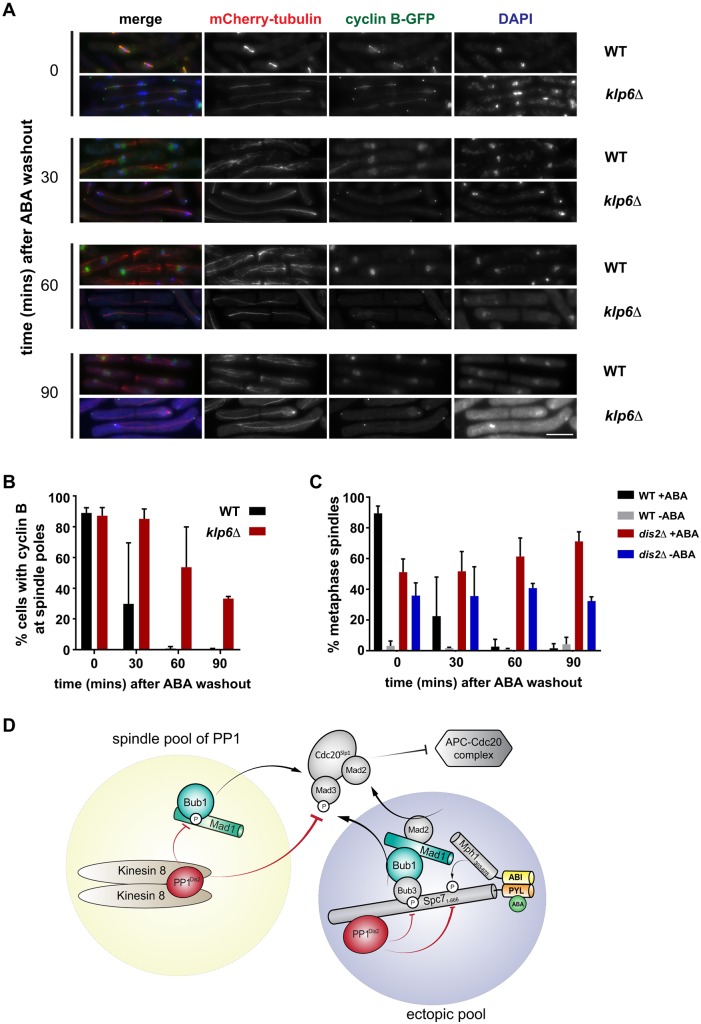
**Checkpoint silencing in SynCheckABA is also slowed when other recruitment sites for PP1 are removed from spindles.** (A) Deletion of kinesin 8 (Klp6) leads to reduced silencing efficiency. Images of cells with and without Klp6 deleted are shown after ABA wash-out (time zero) and 30, 60 and 90 min post-wash. Microtubules are seen in red (mCherry-Atb2), cyclin B in green (Cdc13-GFP) and chromatin in blue (DAPI). Scale bar: 10 µm. (B) Quantification of this release from checkpoint arrest in strains with (WT) and without Klp6 (*klp6Δ*). Cells were scored as arrested if Cdc13-GFP was enriched at spindle poles. This experiment was repeated three times. More than 100 cells were analysed per strain at each time point. (C) *dis2Δ* mutants have profound silencing defects. Quantification of the release from the checkpoint arrest is shown for wild-type and *dis2Δ* cells (plus ABA or DMSO). Cells were scored as metaphase arrested if they had short metaphase spindles and a single mass of condensed chromatin. Results for DMSO controls show that *dis2Δ* cells are generally sick, but that ABA addition induces the SynCheckABA, resulting in elevated levels of metaphase-arrested cells. This arrest persists for >60 m after ABA wash-out as *dis2Δ* cells struggle to silence the checkpoint. This experiment was repeated three times. More than 200 cells were analysed per strain at each time point. See Fig. S4 for fixed cell images from this timecourse. (D) General model of relevant PP1-dependent silencing pathways. The schematic describes two pools of PP1, one that is recruited to the ectopic Spc7-Mph1 signalling scaffold, via the A and B motifs on Spc7, and a second pool that is more generally recruited to the spindle through interaction with kinesin 8 (Klp6). These two pools act together to inhibit MCC-APC/C assembly and thereby enable checkpoint silencing and mitotic exit. All data are plotted as mean±s.d.

Employing SynCheckABA, we tested mutations of the A and B motifs at the N-terminus of Spc7 and removal of the Klp6 kinesin. For these experiments (Figs 3 and 4), all strains contained endogenous wild-type *Mph1* kinase and thus were able to recruit checkpoint proteins to their kinetochores. These include the Mph1 and Bub1 kinases, which are also thought to also have ‘error correction’ functions. Thus, silencing probably needs to take place not only at the ectopic Mph1-Spc7 signalling scaffold, but also at kinetochores. Strains were pre-synchronised in G2 using *cdc25*, released and arrested at metaphase using ABA, and then washed to terminate checkpoint signalling. Progression through anaphase was scored through analysis of spindle elongation and/or cyclin B degradation (using Cdc13-GFP) over a 90 min time course. Mutation of the A motif delayed spindle elongation by 30 min and the A/B double mutant was delayed even more profoundly (Fig. 3B,C). This indicates that PP1 activity on or near the Spc7 protein (previously phosphorylated by Mph1 kinase) is a limiting factor in checkpoint silencing. This system will prove useful for dissecting the regulation of PP1 binding to Spc7 in more detail, and for analysis of putative regulators of PP1 activity.

Mutation of fission yeast kinesin 8 (either Klp5 or Klp6) leads to stabilisation of microtubules, aberrant chromosome movements and long metaphase spindles (Gergely et al., 2016; Klemm et al., 2018; Meadows et al., 2011; West et al., 2002). In these mutants, checkpoint silencing defects cannot simply be analysed through spindle elongation. Instead, we imaged Cdc13-GFP and used the decrease in the number of cells with cyclin B enriched at their spindle poles as a measure of checkpoint silencing. Fig. 4A,B demonstrates that deletion of Klp6 significantly reduces the efficiency of silencing and cyclin B degradation. Finally, we analysed the silencing defect upon deletion of PP1 phosphatase (*dis2Δ*). In the *dis2Δ* strain, which is rather sick, checkpoint silencing was extremely defective with no significant drop in Cdc13-GFP levels over the 90 min time course (Fig. 4C). It should be noted that these *dis2Δ* strains display significant mitotic delays, even in the absence of ABA addition, presumably because the lack of this mitotic phosphatase leads to pleiotropic mitotic defects (see Fig. S4 for images of these cells).

Thus, SynCheckABA neatly recapitulates the balance of opposing kinase and phosphatase activities between Mph1-dependent checkpoint activation and PP1-driven checkpoint silencing on Spc7 and kinesin 8-dependent pathways (see general model in Fig. 4D).

## DISCUSSION

Here, we employed CID to generate a rapid, controlled spindle checkpoint arrest. Addition of ABA to SynCheckABA strains induces the heterodimerisation of Mph1-ABI and Spc7-PYL fusion proteins and this is sufficient to generate an activated signalling scaffold and metaphase arrest within minutes. Like our original SynCheck assay, which was driven by constitutive TetR homodimers (Yuan et al., 2017), this arrest acts independently of spindle checkpoint signalling at endogenous kinetochores, but is dependent on downstream checkpoint components such as Mad1.

A significant advantage of SynCheckABA is that we can wash out the ABA and study the kinetics and mechanism of spindle checkpoint silencing. This was not possible with the original SynCheck strain as we were unable to control TetR dimerisation and thus unable to dissociate the Mph1-TetR–Spc7-TetR signalling scaffold.

Using this new assay, we confirmed that PP1 is crucial for silencing the Mph1-Spc7 scaffold (Figs 3 and 4). PP1 binds to the N-terminus of Spc7, not far from the conserved MELT motifs that, once phosphorylated by Mph1, bind Bub3-Bub1 complexes to initiate generation of the mitotic checkpoint complex (MCC) (Shepperd et al., 2012). Thus, Spc7 acts as the platform for both checkpoint activation and silencing and appears to be a major site of action for both checkpoint activation kinases and silencing phosphatases (Meadows et al., 2011). It is important to note that not all aspects of silencing are recapitulated in our ectopic assay, as some of these relate to specific kinetochore processes that are not captured.

Kinesin 8 is also confirmed as a PP1 recruitment site relevant for checkpoint silencing in SynCheckABA. The phenotypes of the *klp6Δ* mutant suggest that targeting of PP1 to spindle microtubules and kinetochores is also relevant to mitotic exit from an ABA-induced arrest, even though the arrest is initiated away from the kinetochore (see Fig. 4D).

### Advantages of SynCheckABA, over other forms of reconstitution

We believe that all forms of spindle checkpoint reconstitution are useful for mechanistic dissection of this dynamic signalling pathway, whether this be *in vitro* within cytoplasmic extracts (Minshull et al., 1994), *in vitro* with purified recombinant proteins (Faesen et al., 2017) or *in vivo* with synthetically re-wired and simplified signalling pathways (SynCheckABA). The advantages of the latter system are as follows.

(1) The signalling pathway downstream of Spc7 and the downstream effectors are present at normal physiological levels and there are simple, quantitative physiological read-outs (cyclin B degradation, sister chromatid separation and/or anaphase spindle elongation).

(2) Checkpoint arrest is induced in the absence of additional stresses; simple addition of ABA (low toxicity) to the growth media is sufficient for checkpoint activation. There is no need for cold shock (to depolymerise tubulin, *nda3* arrest), heat shock (to perturb temperature-sensitive kinetochore mutants) or overexpression of checkpoint activators.

(3) The PYL and ABI domains have limited cross-reaction in yeast as they are derived from plant proteins. Although we have not compared them directly, we believe that ABA has certain advantages over the use of rapamycin, a very popular CID. To use rapamycin in fission yeast one needs to engineer strains to remove endogenous rapamycin-binding proteins, such as by deleting the *fkh1*+ gene that encodes a native FKBP12 domain (Ding et al., 2014). Importantly, because ABA does not bind tightly to the PYL domain, we can wash ABA out easily to initiate checkpoint silencing. By comparison, rapamycin is very difficult to wash out, making efficient release experiments unrealistic.

(4) Compared to *in vitro* studies with large, recombinant complexes, these fission yeast experiments are simple, cheap and fast. The system also enables rapid iterative studies, because of the ease of further genetic manipulation in yeast.

(5) Importantly, we can easily test candidate regulators (e.g. silencing factors) without needing to know what complexes they are part of, purifying them and worrying about their relevant concentration and post-translational modifications.

(6) Compared with our transcriptionally controlled SynCheck (which employs *nmt-tetR-Mph1*), the ABI-PYL system is less leaky, enabling sick strains (such as the *dis2* mutant analysed in Fig. 4) to be constructed. Previously, we were unable to isolate *nmt-tetR-Mph1,* *dis2Δ* strains because of leaky expression from the weak *nmt81* promoter.

Our ongoing studies with SynCheckABA will enable a detailed mechanistic dissection of PP1-mediated spindle checkpoint silencing in fission yeast. We believe that ABA holds promise as an alternative CID to rapamycin and that it has significant advantages.

## MATERIALS AND METHODS

### P_adh41_-Mph1_303-678_-3xHA-ABI

Mph1 (residues 303-678) was amplified from a pDONR 201 plasmid containing Mph1 (303-678) (Yuan et al., 2017). 3×HA was amplified from a plasmid from the Allshire laboratory (University of Edinburgh) containing codon-optimised PYL-3×HA. ABI was amplified from a pMT_CID_ABI_VS_H vector from the Patrick Heun laboratory (University of Edinburgh). These PCR fragments were treated with Dpn1 and assembled into a Sma1-digested and antarctic phosphatase-treated gel-purified pRad41 yeast expression vector by Gibson assembly.

### P_adh21_-Spc7_1-666_-PYL

The yeast expression vector pIY03 (Yuan et al., 2017) was digested with *Nhe*1 and *Xho*1 and gel purified. The insert (mCherry-2×FLAG-Spc7_1-666_) was used as a template to amplify Spc7_1-666_. PYL was amplified from a bVNI-221 vector from the Heun laboratory. The fragments were then assembled into the digested pIY03 vector backbone using Gibson assembly.

### P_adh21_-spc7_1-666_-mCherry-2×FLAG-PYL (PP1-binding site mutants)

Plasmids containing full-length Spc7 PP1-binding mutants (ΔA, deletion of residues 136–150; ΔAB, deletion of residues 136-150 and residues 331–345) (provided by the Millar laboratory, University of Warwick) were used as templates to amplify mutant versions of Spc7_1-666_. NheI-NLS and PacI sites were introduced during amplification, allowing Spc7 constructs to be digested and ligated into digested pIY03-derived vector backbone, which also contained a C-terminal mCherry-2×FLAG-PYL tag.

### Fission yeast strains

The fission yeast strains used are listed in Table S1.

### *cdc25-22* synchronisation

Cells were grown at 25°C for 1-2 days on YES (rich yeast media, with additional leucine, arginine, lysine, histidine and uracil) plates. They were then pre-cultured in 10 ml of liquid YES containing amino acid supplements at 25°C over the day and inoculated into a larger culture of YES overnight. The following day, log phase cultures were shifted to 36°C for 3.5 h to block in G2. After this, cultures were cooled quickly in iced water to rapidly shift them back to 25°C and release them from the G2 block.

### Synthetic arrest assay

Following a *cdc25-22* block, 250 mM ABA stock (Sigma Aldrich A1049) was added to cultures 5 min after release (20 min if comparing to a carbendazim arrest) to achieve a final concentration of 250 μM (unless otherwise stated).

### Synthetic arrest assay wash-out

Following an ABA-induced synthetic arrest, the cells were washed three times with 50 ml YES.

### Fixing cells and microscopy

Culture (1-1.5 ml) was centrifuged for 1 min at 6000 rpm. The cell pellet was fixed in 200–500 μl of 100% ice-cold methanol. To image cells, 8 μl of the cell suspension in methanol was added to a glass slide; when the methanol evaporated, 1-2 μl DAPI (0.4 μg/ml) was added to the sample and a glass cover slip was placed on top.

Cells were imaged immediately using a 100× oil immersion lens and a Zeiss Axiovert 200M microscope (Carl Zeiss), equipped with a CoolSnap CCD camera (Photometrics) and Slidebook 5.0 software (3i, Intelligent Imaging Innovations). Typical acquisition settings were 300 ms exposure (FITC and TRITC) and 100 ms exposure (DAPI), 2× binning, Z-series over 3 μm range in 0.5 μm steps (seven planes).

### Carbendazim arrest

Following a cdc25-22 block, 3.75 mg/ml stock of carbendazim (Sigma Aldrich) was added to cultures 20 min after release to achieve a final concentration of 100 μg/ml.

### Cell viability assay

Following a synthetic arrest assay, cells from 1 ml of culture were harvested by centrifugation at 6000 rpm for 1 min and re-suspended in 1 ml of distilled water. Tenfold serial dilutions were made in distilled water. Cells were diluted by factors of 100 and 1000, and 0.1 ml plated in triplicate. Colony forming units (cfu) per millilitre of culture was calculated and cell viability over time was plotted as a percentage relative to that at time zero.

## Supplementary Material

Supplementary information
