# Supplementary material for: Regulated reconstitution of spindle checkpoint arrest and silencing through chemically induced dimerisation in vivo
Source: J Cell Sci. 2018 Oct 4;132(4):jcs219766. doi: 10.1242/jcs.219766 (PMC6398473; doi:10.1242/jcs.219766)
Supplement: Supplementary information [file joces-132-219766-s1.pdf]

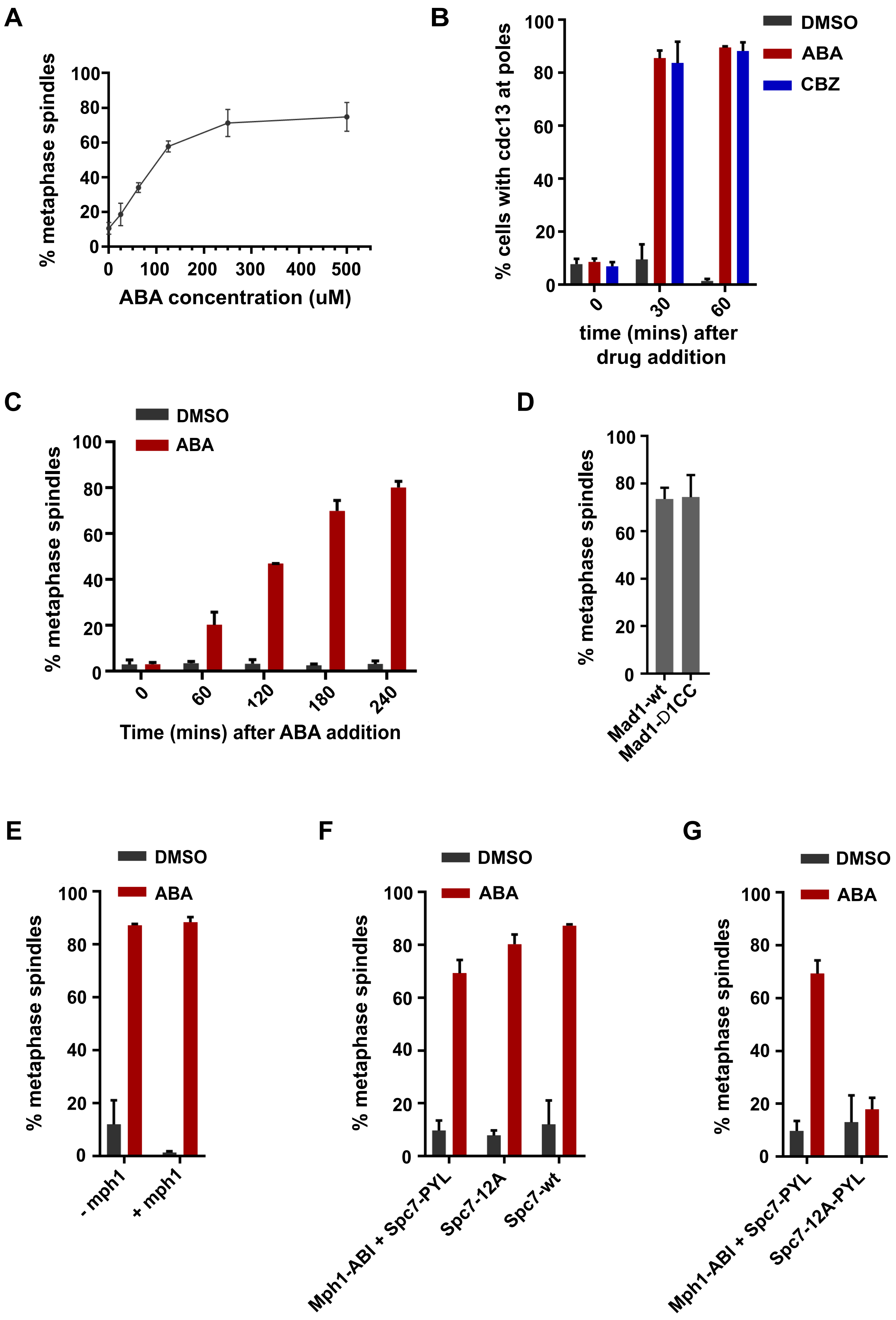

Figure S1 Amin et al

Journal of Cell Science • Supplementary information

Figure S1

(A) The effect of ABA concentration on Mph1-ABI Spc7-PYL driven arrest. Different concentrations (0, 25, 62.5, 125, 250, 500 μM) of ABA were used to induce arrest in cultures 5 mins after release from the G2 block. Samples were fixed at 60 mins and scored as metaphase arrested if they had short metaphase spindles and a single mass of condensed chromatin. >100 cells were analysed per strain at each time point. This experiment was repeated 3 times. Data plotted as mean +/- SD.

(B) Quantification of SynCheckABA strain containing Cdc13-GFP at 60 minutes after treatment with either DMSO, ABA or CBZ (added 20 minutes after release from G2 block). Cells were scored as arrested if Cdc13 was enriched at spindle poles and/or if short metaphase spindle was present. >240 cells were analysed per strain at each time point. This experiment was repeated 2 times. Data plotted as mean +/- SD.

(C) Quantification of Mph1-ABI Spc7-PYL cultures (plus ABA or DMSO) through a 4 hour time course at 25°C without synchronisation in G2. Samples were fixed every 60 minutes and scored as metaphase arrested if they had short metaphase spindles and a single mass of condensed chromatin. >250 cells were analysed per strain at each time point. This experiment was repeated 2 times. Data plotted as mean +/- SD.

(D) Quantification of Mph1-ABI Spc7-PYL arrest in strains with and without the N-terminal coiled-coil of Mad1, 60 minutes after release from G2 block (ABA added at 5 minutes). In its absence, Mad1p fails to localise to the nuclear periphery or bind to the Cut7 kinesin which would otherwise take it to spindle poles. >150 cells were analysed per strain at each time point. This experiment was repeated 3 times. Data plotted as mean +/- SD.

(E) Quantification of ABA-induced arrest in strains with and without endogenous mph1 60 minutes after release from G2 block (ABA added at 5 minutes). >100 cells were analysed per strain at each time point. This experiment was repeated 2 times. Data plotted as mean +/- SD.

(F) Quantification of the strains indicated at the 60 minute time point after release from the G2 block (ABA added 5 mins after release). The spc7-12A strain has the endogenous spc7 gene deleted and expresses this non-phosphorylatable 12A (MELA) allele from its own promoter integrated at the C locus. Spc7-wt is the wild-type control for this strain. > 100 cells were analysed per strain at each time point. This experiment was repeated 3 times. Data plotted as mean +/- SD.

(G) Quantification of the strains indicated at the 60 minute time point after release from the G2 block (ABA added 5 mins after release). Spc7-12A-PYL is a non-phosphorylatable 12A (MELA) mutant tagged with PYL, which does not generate an arrest when co-recruited with Mph1-ABI in the presence of ABA. >220 cells were analysed per strain at each time point. This experiment was repeated ≥ 2 times. Data plotted as mean +/- SD.

Journal of Cell Science • Supplementary information

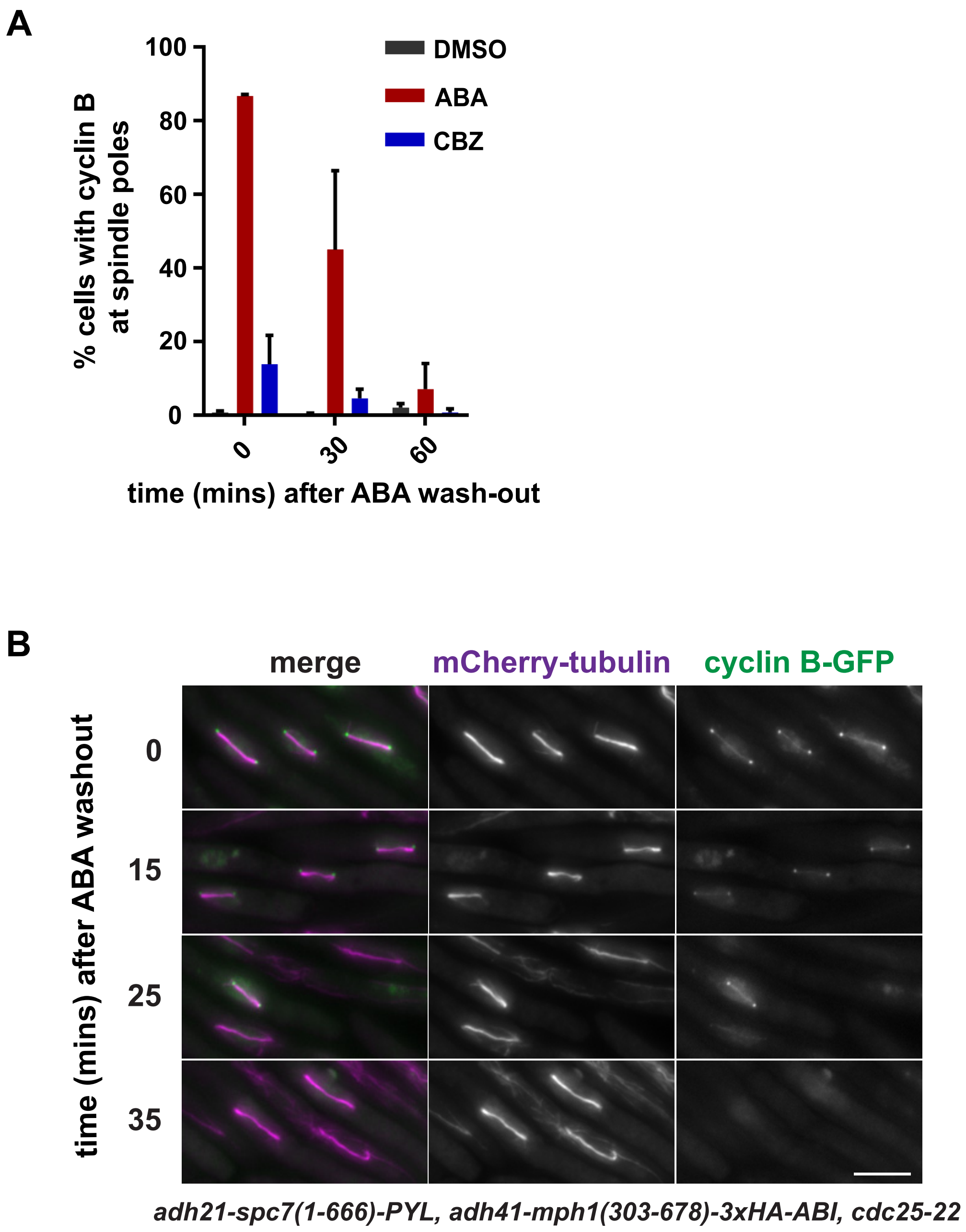

(A) Quantification of DMSO/ABA/CBZ wash-out in SynCheckABA strain following 60 minutes of treatment with solvent (added 20 minutes after release from G2 block).
Cells were scored as arrested if Cdc13 was enriched at spindle poles.
>160 cells were analysed per strain at each time point.
This experiment was repeated 2 times and data are plotted as mean +/- SD.

(B) Fixed cell images taken of the arrested SynCheckABA strain at 0, 15, 25 and 35 minutes after ABA wash-out. Microtubules are seen in purple (mCherry-tubulin) and cyclin B in green (cyclin B-GFP). Scale bar is 10 μm.

Figure S2 Amin et al

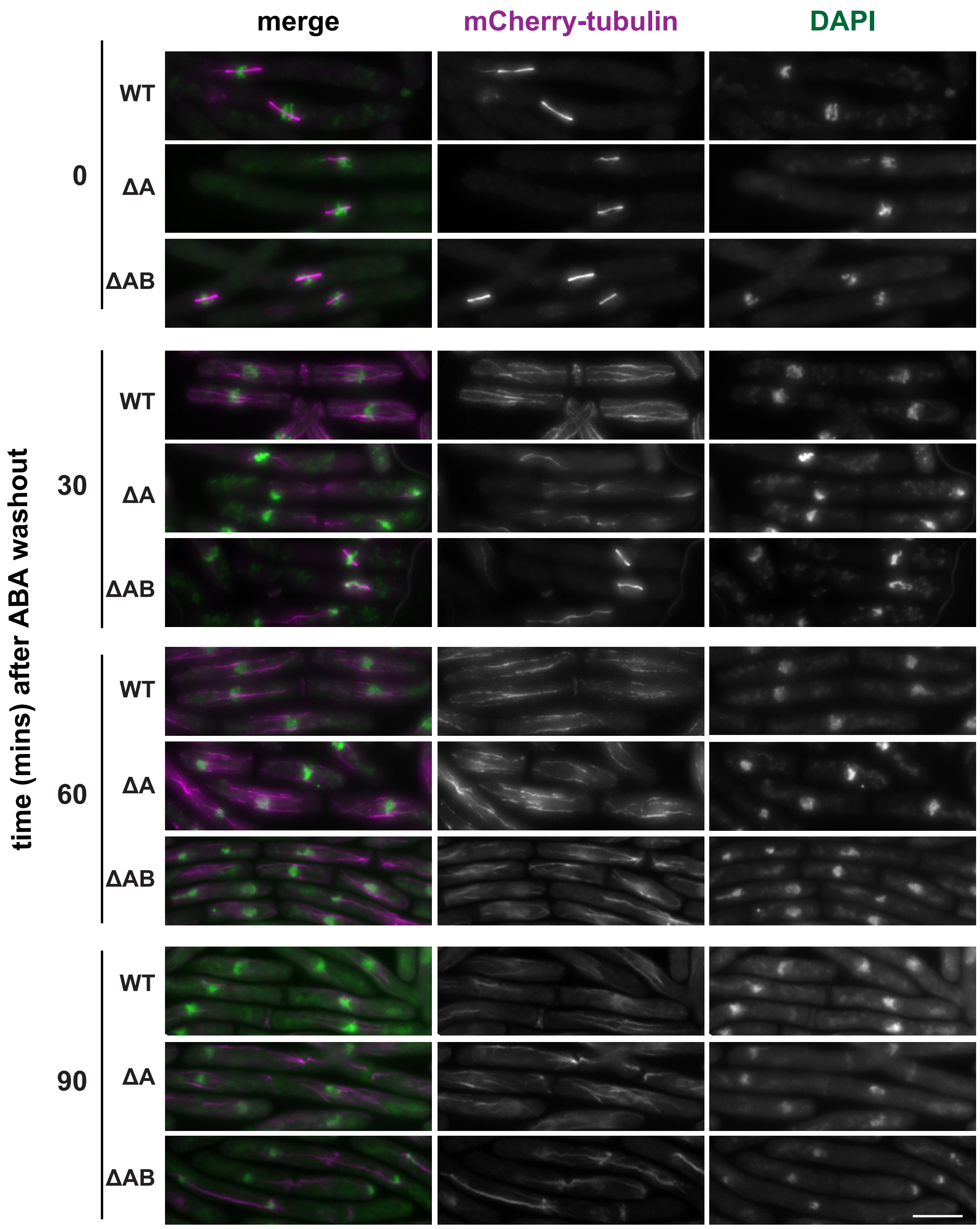

Fixed cell images taken of the arrested Spc7 mutant strains at 0, 15, 25 and 35 minutes after ABA wash-out. Microtubules are seen in purple (mCherry-tubulin) and DAPI in green. Scale bar is 10 µm.

Figure S3 Amin et al

Journal of Cell Science • Supplementary information

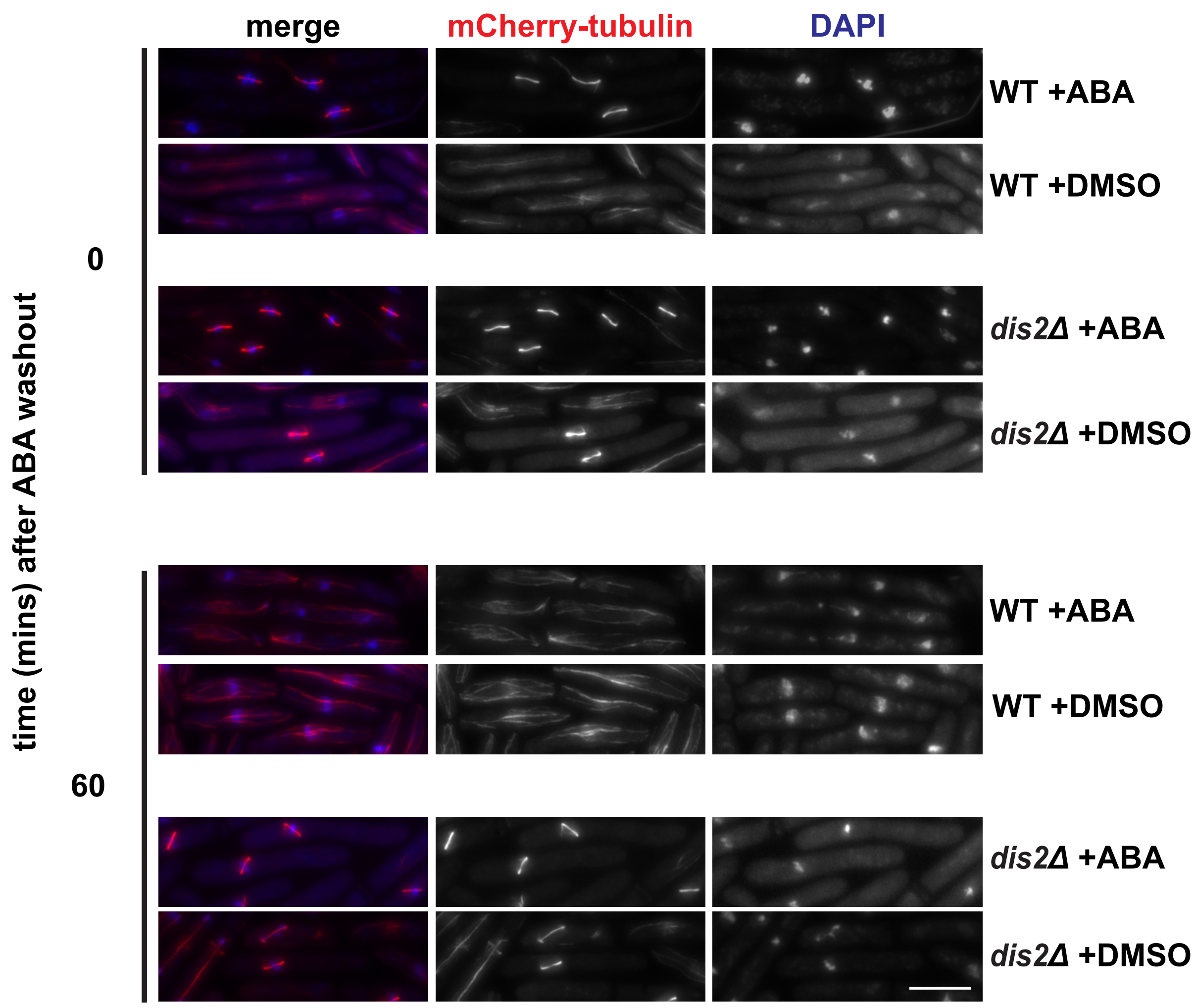

*dis2*Δ mutants have profound silencing defects. Fixed cell images of wild-type and *dis2*Δ cells after ABA washout (time zero) and 60 minutes post-wash. Microtubules are seen in red (mCherry-tubulin) and chromatin in stained with DAPI. Scale bar is 10 µm.

Figure S4 Amin et al

Supplementary Table 1

Figure	Strain	Genotype
Figure 1	PA252	Padh41-mph1(303-678)-3xHA-ABI:LEU2 mph1Δ::nat lys1::Padh21-spc7(1-666)-PYL:ura4 cdc25-22 Z:Padh15-mCherry-atb2:natMX6 bub1-GFP:his
	PA269	Padh41-mph1(303-678)-3xHA-ABI:LEU2 mph1Δ::nat cdc25-22 Z:Padh15-mCherry-atb2:natMX6 bub1GFP:his
	PA286	lys1::Padh21-spc7(1-666)-PYL:ura4 cdc25-22 Z:Padh15-mCherry-atb2:natMX6 bub1-GFP:his
	PA260	mad1Δ::hyg Padh41-mph1(303-678)-3xHA-ABI:LEU2 mph1Δ::nat lys1::adh21-spc7(1-666)-PYL:ura4 cdc25-22 Z:Padh15-mCherry-atb2:natMX6; bub1-GFP:his
	PA338	Padh41-mph1(303-678)-3xHA-ABI:LEU2 lys1::Padh21-spc7(1-666)-PYL:ura4 cdc25-22 Z:Padh15-mCherry-atb2:natMX6 cdc13-GFP:leu
Figure 2	PA338	Padh41-mph1(303-678)-3xHA-ABI:LEU2 lys1::Padh21-spc7(1-666)-PYL:ura4 cdc25-22 Z:Padh15-mCherry-atb2:natMX6 cdc13-GFP:leu
	PA252	Padh41-mph1(303-678)-3xHA-ABI:LEU2 mph1Δ::nat lys1::Padh21-spc7(1-666)-PYL:ura4 cdc25-22 Z:Padh15-mCherry-atb2:natMX6 bub1-GFP:his
Figure 3	SS123	Padh41-mph1(303-678)-3xHA-ABI:LEU2 lys1::Padh21-spc7(1-666)-mCherry-2xFLAG-PYL:ura4 cdc25-22 Z:Padh15-mCherry atb2:natMX6
	SS121	Padh41-mph1(303-678)-3xHA-ABI:LEU2 lys1::Padh21-spc7(1-666, Δ136-150)-mCherry-2xFLAG-PYL:ura4 cdc25-22 Z:Padh15-mCherry-atb2:natMX6
	SS122	Padh41-mph1(303-678)-3xHA-ABI:LEU2 lys1::Padh21-spc7(1-666, Δ136-150, Δ331-345)-mCherry-2xFLAGPYL:ura4 cdc25-22 Z:Padh15-mCherry-atb2:natMX6
Figure 4	PA338	Padh41-mph1(303-678)-3xHA-ABI:LEU2 lys1::Padh21-spc7(1-666)-PYL:ura4 cdc25-22 Z:Padh15-mCherry-atb2:natMX6 cdc13-GFP:leu
	SS130	klp6Δ::ura4 Padh41-mph1(303-678)-3xHA-ABI:LEU2 lys1::Padh21-spc7(1-666)-mCherry-2xFLAG-PYL:ura4 cdc25-22 Z:Padh15-mCherry-atb2:natMX6 cdc13-GFP:leu
	SS123	Padh41-mph1(303-678)-3xHA-ABI:LEU2 lys1::Padh21-spc7(1-666)-mCherry-2xFLAG-PYL:ura4 cdc25-22 Z:Padh15-mCherry-

Journal of Cell Science • Supplementary information

		atb2:natMX6
	SS128	dis2Δ::hyg Padh41-mph1(303-678)-3xHA-ABI:LEU2 lys1::Padh21-spc7(1-666)-mCherry-2xFLAG-PYL:ura4 cdc25-22 Z:Padh15-mCherry-atb2:natMX6 cdc13-GFP:leu
Figure S1	PA252	Padh41-mph1(303-678)-3xHA-ABI:LEU2 mph1D::nat lys1::Padh21-spc7(1-666)-PYL:ura4 cdc25-22 Z:Padh15-mCherry-atb2:natMX6 bub1-GFP:his
	PA338	Padh41-mph1(303-678)-3xHA-ABI:LEU2 lys1::Padh21-spc7(1-666)-PYL:ura4 cdc25-22 Z:Padh15-mCherry-atb2:natMX6 cdc13-GFP:leu
	PA253	Padh41-mph1(303-678)-3xHA-ABI:LEU2 mph1D::nat lys1::Padh21-spc7(1-666)-PYL:ura4 2xflag-mad1-Δ1CC:hyg cdc25-22 Z:Padh15-mCherry-atb2:natMX6 bub1-GFP:his
	PA 254	Padh41-mph1(303-678)-3xHA-ABI:LEU2 mph1D::nat lys1::Padh21-spc7(1-666)-PYL:ura 2xflag-mad1-Δ1CC:hyg cdc25-22 Z:Padh15-mCherry-atb2:natMX6 bub1-GFP:his
	PA264	spc7Δ::G418 spc7:hyg lys1::Padh21-spc7(1-666)-PYL:ura4 Padh41-mph1(303-678)-3xHA-ABI:LEU2 mph1Δ::nat cdc25-22 Z:Padh15-mCherry-atb2:natMX6
	PA262	spc7Δ::G418 spc7-T12A:hyg lys1::Padh21-spc7(1-666)-PYL:ura4 Padh41-mph1(303-678)-3xHA-ABI:LEU2 mph1Δ::nat cdc25-22 Z:Padh15-mCherry-atb2:natMX6
	PA317	Padh41-mph1(303-678)-3xHA-ABI:LEU2 lys1::Padh21-spc7(1-666)12A-PYL:ura4 cdc25-22 Z:Padh15-mCherry-atb2:natMX6
Figure S2	PA338	Padh41-mph1(303-678)-3xHA-ABI:LEU2 lys1::Padh21-spc7(1-666)-PYL:ura4 cdc25-22 Z:Padh15-mCherry-atb2:natMX6 cdc13-GFP:leu
Figure S3	SS123	Padh41-mph1(303-678)-3xHA-ABI:LEU2 lys1::Padh21-spc7(1-666)-mCherry-2xFLAG-PYL:ura4 cdc25-22 Z:Padh15-mCherry atb2:natMX6
	SS121	Padh41-mph1(303-678)-3xHA-ABI:LEU2 lys1::Padh21-spc7(1-666, Δ136-150)-mCherry-2xFLAG-PYL:ura4 cdc25-22 Z:Padh15-mCherry-atb2:natMX6
	SS122	Padh41-mph1(303-678)-3xHA-ABI:LEU2 lys1::Padh21-spc7(1-666, Δ136-150, Δ331-345)-mCherry-2xFLAGPYL:ura4 cdc25-22 Z:Padh15-mCherry-atb2:natMX6
Figure S4	SS128	dis2Δ::hyg Padh41-mph1(303-678)-3xHA-ABI:LEU2 lys1::Padh21-spc7(1-666)-mCherry-2xFLAG-PYL:ura4 cdc25-22 Z:Padh15-mCherry-atb2:natMX6 cdc13-GFP:leu
	SS123	Padh41-mph1(303-678)-3xHA-ABI:LEU2 lys1::Padh21-spc7(1-666)-mCherry-2xFLAG-PYL:ura4 cdc25-22 Z:Padh15-mCherry-atb2:natMX6

Journal of Cell Science • Supplementary information
